# Finite Element Study of Stress Distribution with Tooth-Supported Mandibular Overdenture Retained by Ball Attachments or Resilient Telescopic Crowns

**DOI:** 10.1055/s-0042-1749363

**Published:** 2022-11-09

**Authors:** Nour M.T. Ajaj AL-Kordy, Mohannad H. AL-Saadi

**Affiliations:** 1 Department of Removable Prosthodontics, Faculty of Dental Medicine, Damascus University, Damascus, Syria

**Keywords:** finite element analysis, ball attachment, telescopic crowns, overdenture

## Abstract

**Objective** The removable partial denture must keep health of the remaining teeth and the supporting tissues through the distribution of chewing forces on the abutment teeth and alveolar process.

This study aimed to evaluate stress distribution with canines-supported mandibular overdenture retained by two different attachment types: ball attachments or resilient telescopic crowns.

**Materials and Methods** Two 3-dimensional finite element models consisting of the cortical mandible bone, cancellous mandible bone, oral mucosa, canines, periodontal ligaments, the two attachment types, and overdenture were simulated. The models were imported into the mathematical analysis software Ansys Workbench V 15.0. All materials were considered to be homogeneous, isotropic, and linearly elastic. A vertical bilateral load of 120 N was applied to the central fossa of the first molars. The von Mises stress was calculated for canines, cortical, and cancellous bone.

**Results** The maximum von Mises stress of the ball attachments model was 35.61, 4.28, 7.82, and 1.29 MPa for canines, cortical alveolar bone of canines, cortical alveolar bone at the distal end of the overdenture, and cancellous alveolar bone of canines, respectively. The maximum von Mises stress of the resilient telescopic crowns model was 39.22, 4.74, 7.06, and 1.05 MPa for canines, cortical alveolar bone of canines, cortical alveolar bone at the distal end of the overdenture, and cancellous alveolar bone of canines, respectively.

**Conclusion** Resilient telescopic crowns distribute the stresses between canines, alveolar bone of canines, and overdenture supporting alveolar bone. Ball attachments transfer less stress to the canines and cortical alveolar bone of the canines, but more stress to the cancellous alveolar bone of canines and alveolar bone at distal end of the overdenture. Resilient telescopic crowns are preferred over ball attachment when the abutment teeth have good periodontal support.

## Introduction

Tooth-supported overdenture is an effective treatment modality for aged patients with few remaining teeth. 1 The benefits of this treatment include alveolar bone maintenance, proprioceptive feedback, retention and stability improvement, and relatively reasonable costs. 2 The selection of attachment system depends on the number, alignment, and periodontal status of the remaining teeth. This selection may be affected by available vertical and horizontal space, cost effectiveness, and skills of dentist. 1 3 4

Ball and socket attachment is used widely. This system can be applied with root and implant-supported prostheses. It is cost effective, easy to apply, and chairside time effective. This attachment system consists of a metal ball attached to the abutment tooth and a matrix component attached to the prosthesis, fitted to the patrix ball, and retained by frictional mechanism. 5 Ball attachment provides vertical and hinge movements. 6

Telescopic overdenture is a prosthesis that includes a primary crown cemented to the abutment and a secondary crown attached to the prosthesis and fits on the primary crown. 1 7 Three types of telescopic crowns have been described: the parallel-walled crowns, the conical crowns with tapered design, and the resilient crowns with clearance fit. 3 8 Clearance fit means free space between primary and secondary crowns. 8 Telescopic crowns provide guidance and stability, prevent dislodging motion of denture, and transfer forces along the axis of the abutment teeth. 4 7 8 The freedom of vertical or rotational movement in resilient telescopic crowns designs provides resilient relation between the abutment and the alveolar mucosa supported the denture, prevents deleterious effect, and prolongs abutment survival 7 9 ; thus, resilient telescopic crowns-retained overdentures are indicated to patients with few remaining teeth. 7 8 9

Removable partial denture (RPD) should keep health and survival of abutment teeth and supporting tissues so that the forces applied to abutment teeth and their effect must be taken into account when designing and constructing the RPD. 10 McCracken emphasized the importance of the distribution of forces on the supporting tissues by providing retention and stability of the RPD when he established biomechanics principles for the design of RPDs. 11 Overdenture is an optimal biomechanical modality of treatment as it allows distribution of chewing forces on the mucosa and alveolar process, in addition forces applied to shortened teeth are more axial. 12

Finite element analysis (FEA) is an important tool for the simulation and prediction of the biomechanical behavior of various types of prosthetic structures in oral environment, such as removable and fixed prosthesis, dental implant, and evaluating integrity at the bone. 13 14 FEA is used to analyze distribution of stress in the components of dental prostheses and their supporting structures, and to study factors that affect the biomechanics of RPDs, such as design of retention, occlusal rest position, design of major connectors, splinting of the abutment teeth, and the use of implant approach. 15 It has been widely used in implant dentistry. The influence of many factors on the biomechanical behavior of implants has been studied, such as implant design, properties of implant material, number and size (length, diameter) of implants, quality and quantity of surrounding bone, and implantation surgical technique. 16 FEA is based on finding a solution to a complex physical problem by dividing a geometric model into a finite number of elements, in which the filed variables can be interpolated with the involvement of specific physical properties and geometric functions. 17 18

FEA consists of three principle steps: preprocessing, processing, and postprocessing.

- Preprocessing: The objective of this step is the constructing of the “model” that consists of the geometrical construction, meshing, the definition of material properties, and boundary conditions. 18
- Processing or solution: This is the step in which the computer software runs the mathematical solution process. 19
- Postprocessing: The results are presented in this step, then verification and conclusions are made. 19

Modeling the geometry can be done using computer-aided design (CAD) software, but often the geometry model needs modifications and simplifications to get a more robust and simple model that is easy to understand and analyze. 19 20 The anatomic geometry can be obtained from different sources such as literature data, three-dimensional (3D) scanners, and computer tomography (CT). 21 There are three types of 3D scanners: laser, light-emitting diode light, and contact. The obtained data are typically recorded in a STL file (“Standard Triangle Language” or “Standard Tessellation Language”). 21 Solid models have been created from data sets of CT. 22 CT and cone beam CT (CBCT) imaging data are obtained in the universal format for “Digital Imaging and Communication in Medicine” (DICOM-format). 23 Data are exported to image-processing software, such as Mimics (Materialise, Leuven, Belgium), 24 where 3D surface models of CT or CBCT data are constructed using segmentation. 23 Segmentation is the isolation of a specific anatomical structure from surrounding structures based on a limited range of grayscale values and exporting it to the virtual 3D model in STL file format. 23 25 Model in STL format is the 3D-surface geometry described in a triangular mesh. 21 The 3D-surface geometry obtained by segmentations results in ribbed surfaces with irregularities and possible gaps. 22 The STL mesh has insufficient quality, and is marked by triangles with damaged edges, so it directs the construction of the geometric model, but not the FEA mesh. 12 21 A program that handles these polygons and constructs solid CAD bodies is needed, such as 3Matic (Materialise). 12 22 26 For reconstruction of model in such a software, the connections between different objects are precisely defined to ensure that there are common nodes between different objects at the communication surfaces. 22 This provides a realistic simulation of load distribution within the structure and a file type that classical CAD systems can process. 12 22 26 CAD software allow the integration of geometry files (e.g., .iges, .step) for high-definition structures, such as surgical plates, dental implants, prosthetics, denture, and restorative materials. 22

The aim of this study was to evaluate stress distribution with canines-supported mandibular overdenture retained by two types of attachments (ball attachments or resilient telescopic crowns) of the canines, cortical alveolar bone, and cancellous alveolar bone using FEA method.

## Materials and Methods

Ethical approval was obtained from Damascus University, and it complies with the Declaration of Helsinki 1975, as revised in 2008. Informed consent was obtained from the patient to participate in this study.

**Fig. 1:**
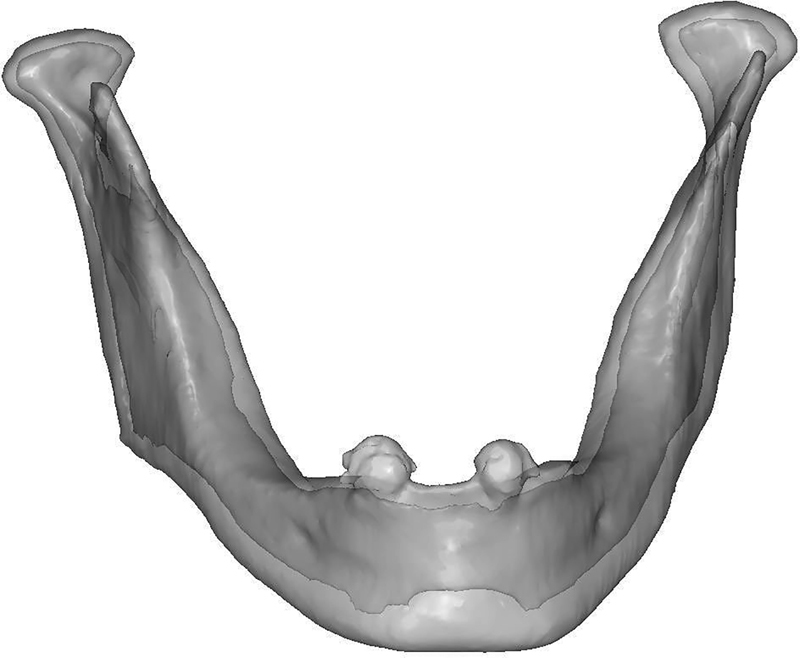
Model 1: mandible model that consists of cortical bone, cancellous bone, and two canine crowns.

**Fig. 2:**
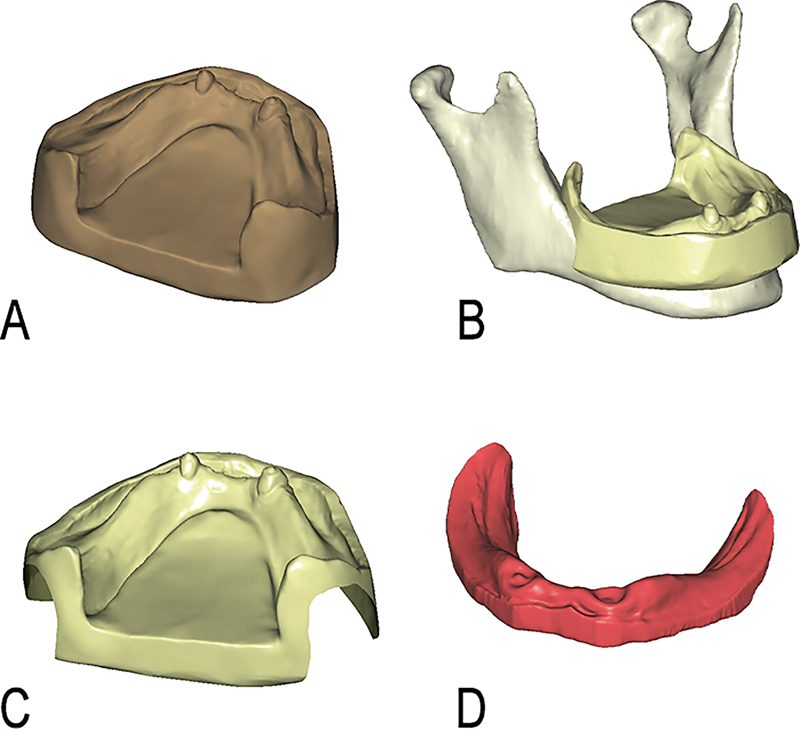
Modeling of mucosa: model 2 ( **A** ); alignment of models 1 and 2 ( **B** ); subtracted model ( **C** ); model 3 ( **D** ).

**Fig. 3:**
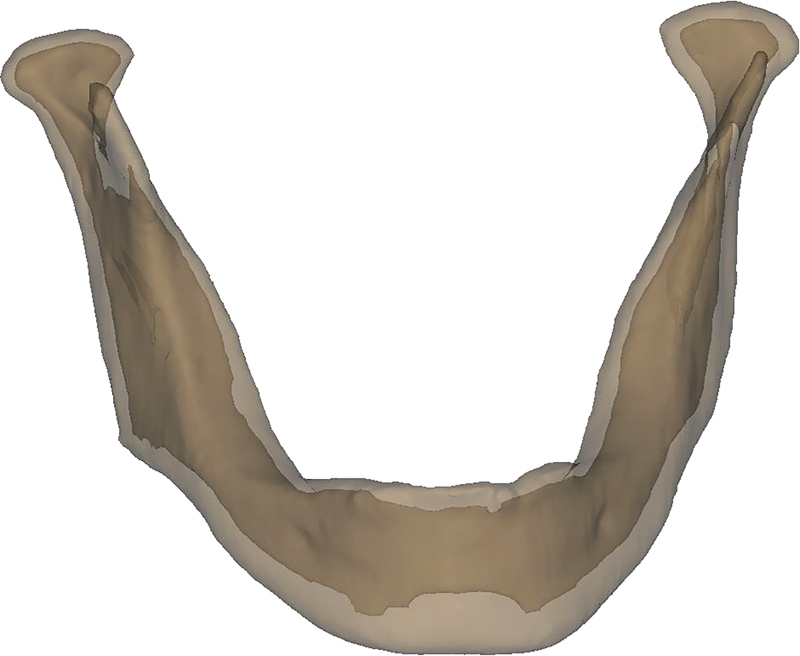
Model 4: final mandible model that consists of cortical bon and cancellous bone.

A 3D FEA solid model of mandible was constructed using CBCT data from a 63-year-old patient with canines-supported mandibular overdenture retained by ball attachments. The mandible was scanned with CBCT machine (Sirona GmbH, Bensheim, Germany) at a 0.25-mm slice thickness. The obtained images were imported into two image-processing software (Mimics 7.3, Materialise) and (3Matic 2, Materialise) for 3D FEA solid model construction. By using grayscale the cortical bone was separated from the cancellous bone of the mandible, thus constructed mandible model (model 1) was consisted of cortical bone, cancellous bone, and two canine crowns, as illustrated in Fig. 1 . The features of oral mucosa was modeled by scanning patient master mandibular model. 14 The mandibular impression was obtained by patient overdenture with light body rubber impression material, and the master model was made of plaster. The model was scanned by desk scanner (3Shape D2000, Copenhagen, Denmark). Obtained data were imported to CAD software (Exocad Dental-CAD, v 3.0; exocad GmbH, Darmstadt, Germany) for surface reconstruction. Smooth master model was obtained (model 2). Model 1 and model 2 were aligned in Exocad by finding the matched points of the two models. The crowns of canines served as matched points of the two models. Model 1 was subtracted from aligned models, and the canines crowns were removed to obtaining the mucosa model (model 3). These procedures are illustrated in Fig. 2 . The canine crowns of model 1 were removed to obtain final mandible model (model 4) as illustrated in Fig. 3 . Models of mandibular canines were designed with Exocad and prepared to receive ball attachments in one model and resilient telescopic crowns in the other. The models of canines were inserted in the mandible in the place of the patient's removed canines, through subtraction Boolean operations in Exocad. The periodontal ligaments (PDLs) of canines were modeled by adding a 0.25-mm thick shell to the contiguity surface between bone and canines models, the thickness of shell was subtracted from the bone. 14 The commercial ball attachment (OT Cap, Rhein 83 Srl, Bologna, Italy) measuring 2.5 mm in diameter was simulated with Exocad. Cap and core fitted to the canine was simulated with Exocad. The male component was attached to the cap and core as one piece bonded to the canine, and the female component was included in the overdenture acrylic resin base. The resilient telescopic crowns were simulated with Exocad. The primary crown was designed with a taper of 6 degrees height of 5 mm. The secondary crown was designed with an occlusal free space of 0.4 mm between the primary and secondary crowns and a tiny amount of circumferential space of 0.04 mm between the two crowns, so vertical movement between the two crowns is allowed. 9 27 The overdenture (assumed to be acrylic resin) model was obtained by scanning the patient overdenture with desk scanner. Obtained data were imported to Exocad for surface reconstruction.

**Fig. 4:**
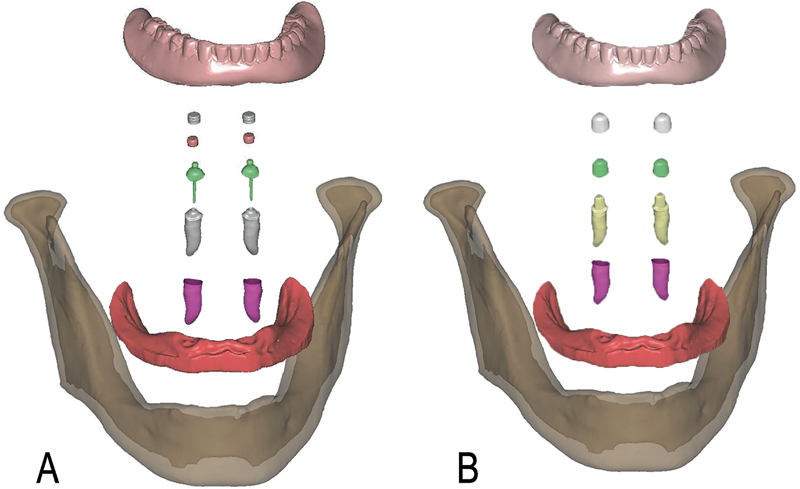
Components of final finite element analysis (FEA) models: the model with ball attachments ( **A** ); the model with resilient telescopic crowns ( **B** ).

**Fig. 5:**
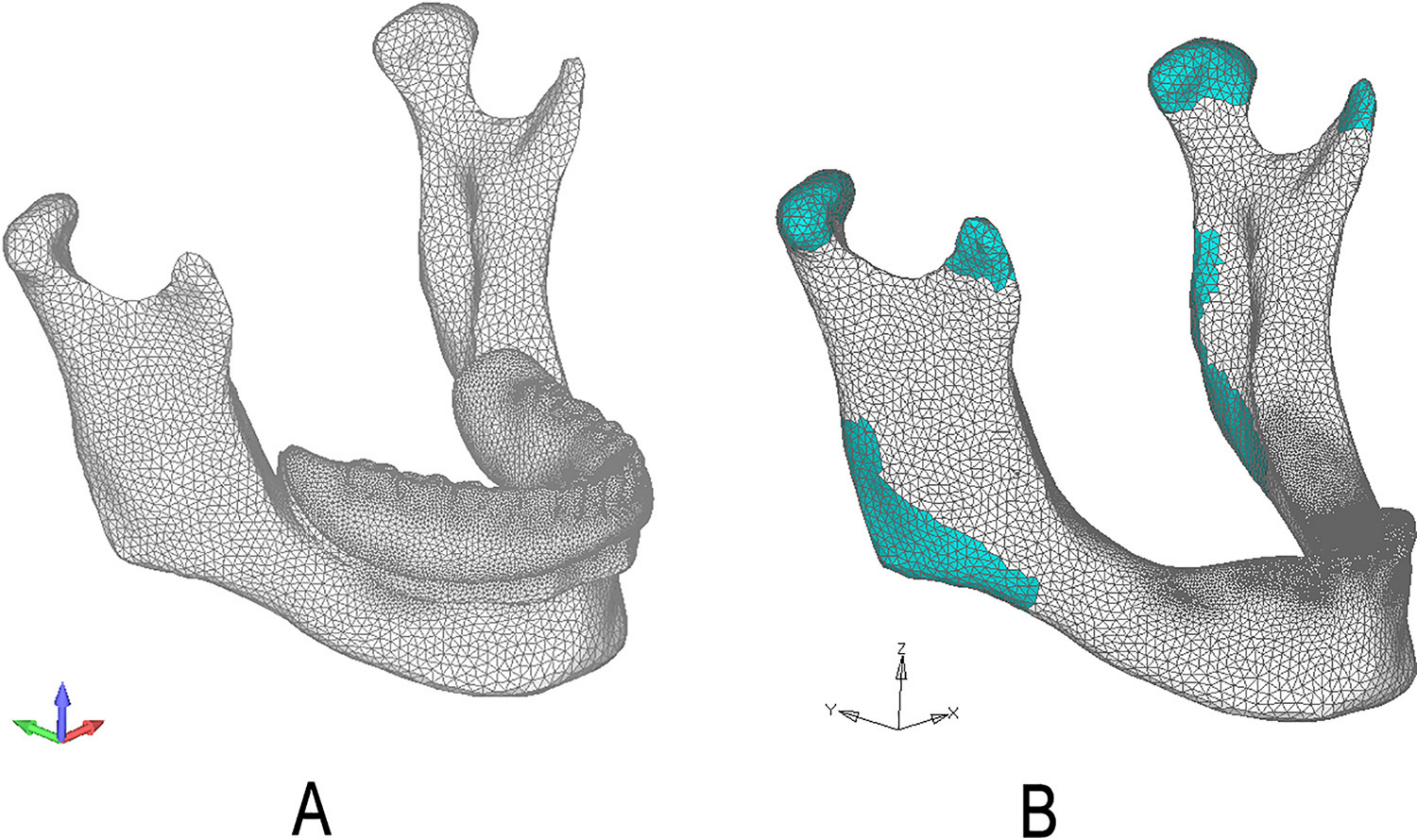
Meshing and fixation of the finite element analysis (FEA) model: the meshed FEA model ( **A** ); the fixation areas of the mandible model ( **B** ).

The models of all structures were imported to analysis software (ANSYS Workbench v15.0; ANSYS Inc) and assembled. The two obtained FEA models consisted of cortical mandible bone, cancellous mandible bone, oral mucosa, canines, PDLs, overdenture, and two types of attachments (ball attachment in one model and resilient telescopic crowns in the other). The components of the final FEA models are illustrated in Fig. 4 . The FEA models were analyzed with ANSYS by meshing models, defining material properties, applying boundary conditions and loading, and finally, obtaining a mathematical solution. A mesh was generated, the 10-node tetrahedral type of element which is recommended for complex geometries was selected to mesh the models. 28 Fig. 5 shows the meshed FEA model. Table 1 shows the number of elements and nodes of the two FEA models. All components were considered homogeneous, isotropic, and linearly elastic. Table 2 shows the elastic modulus and the Poisson ratio for each material with reference to the previous studies. The surface contact between overdenture and mucosa was defined as contact with friction (coefficient of friction 0.334). 29 The contact between matrix and patrix of the ball attachment was defined as contact with friction (coefficient of friction 0.4). 29 The contact between the primary and secondary resilient telescopic crowns was defined as (no separation), that allows the sliding between surfaces without separation. The contact between all the other parts was defined as “bonded.”

**Table 1:** Number of nodes and elements of meshed models

Finite element model	Ball attachments model	Resilient telescopic crowns model
Number of nodes	172,035	155,602
Number of elements	957,418	876,449

**Table 2:** Mechanical properties of materials

Component	Material	Elastic modulus (MPa)	Poisson ratio
Overdenture 29	Acrylic resin	8300	0.28
Mucosa 29	Mucosa	2.8	0.40
Tooth 14	Dentin	18600	0.30
Periodontal ligament 13 30	Periodontal ligament	68.9	0.45
Cortical bone 14	Cortical bone	13700	0.30
Cancellous bone 14	Cancellous bone	1370	0.30
Resilient telescopic crowns 24	Co-Cr alloy	218000	0.33
Metal housing of ball attachment 31	Stainless steel	210000	0.33
Retentive cap of ball attachment 24 28	Nylon rubber	5	0.45
Ball& (cap and core) 24	Co-Cr alloy	218000	0.33

To simulate an occlusal force, a vertical bilateral load of 120 N was applied to the central fossa of first molars. 14 32 The mandible models were fixed from the attachment areas of lateral pterygoid, temporalis, medial pterygoid, masseter muscles, and from where the condylar process joints the temporomandibular articular. 28 29 Fig. 5 shows the fixation areas of the mandible model. Linear static analysis was performed, the von Mises stresses of canines, cortical, and cancellous bone were calculated.

## Results

**Fig. 6:**
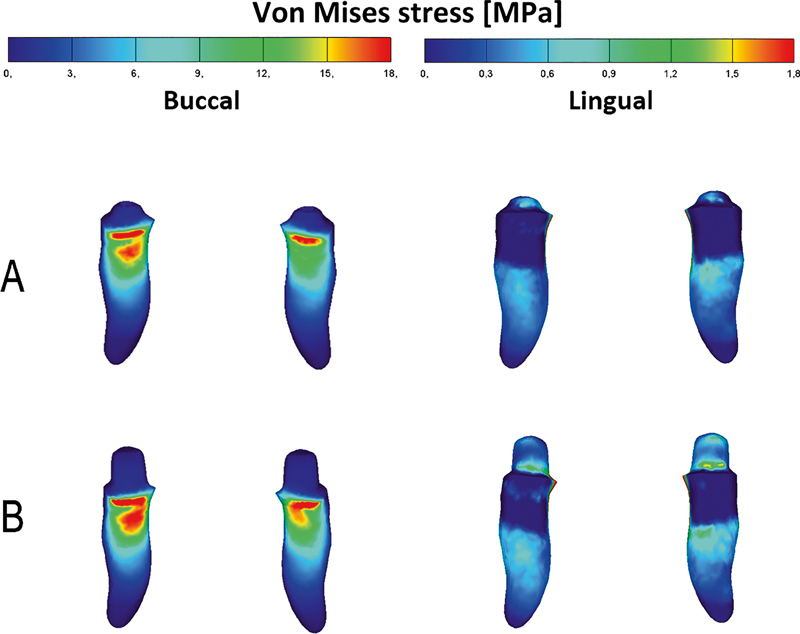
The von Mises stress distribution in canines: ball attachments model ( **A** ); resilient telescopic crowns model ( **B** ).

The von Mises stress fields were obtained in the form of color-coded contour maps. Fig. 6 shows that von Mises stresses of the canines of the two models were similarly distributed. The von Mises stress concentration areas were at the buccal side of the cervical region of the canine roots.

**Fig. 7:**
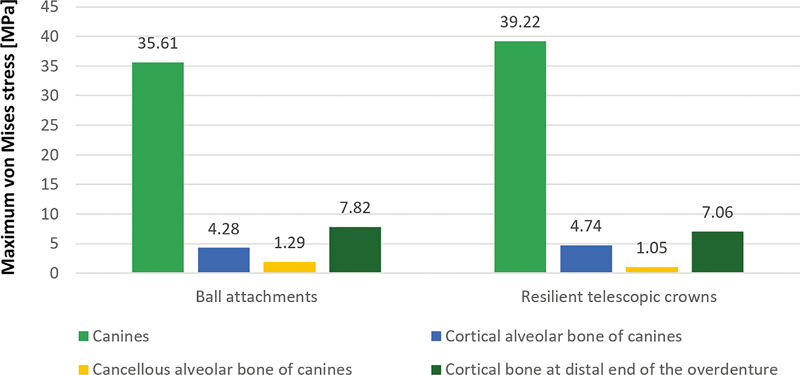
Maximum von Mises stress comparison of canines, cortical, and cancellous bone of the two models under vertical bilateral loading.

As illustrated in Fig. 7 , the maximum von Mises stress of the canines of the ball attachments and resilient telescopic crowns models was 35.61 and 39.22 MPa, respectively.

**Fig. 8:**
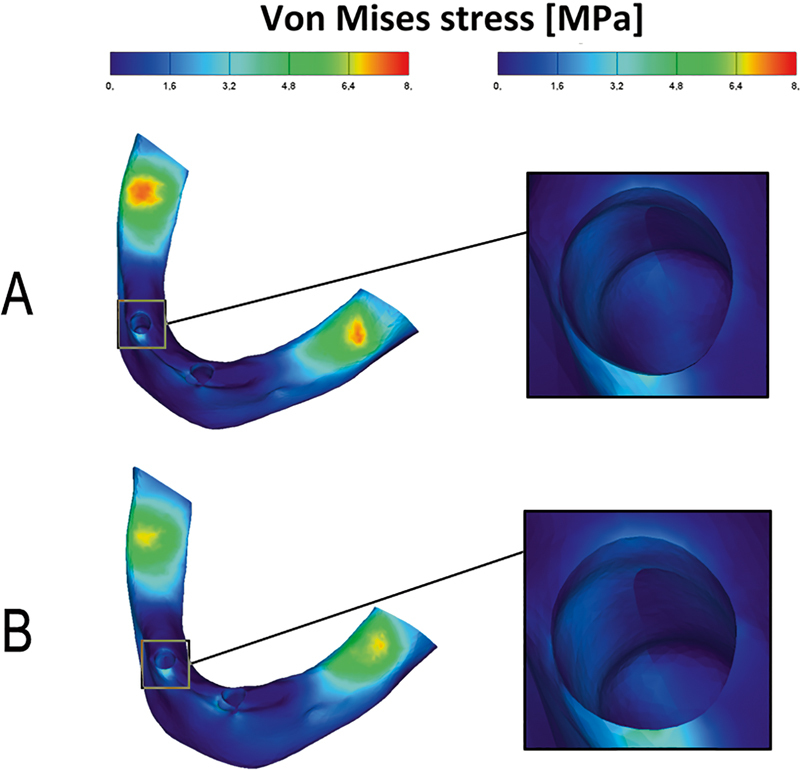
The von Mises stress distribution in the cortical bone: ball attachments model ( **A** ); resilient telescopic crowns model ( **B** ).

Fig. 8 shows that von Mises stresses of the cortical bone of the two models were similarly distributed. The von Mises stress concentration areas were around the canines and at the distal end of the overdenture. The maximum von Mises stress-bearing area of the cortical alveolar bone of the canines was located at the buccal side of the cervical region of the alveolar bone.

As illustrated in Fig. 7 , the maximum von Mises stress of the cortical alveolar bone of the canines of the ball attachments and the resilient telescopic crowns models was 4.28 and 4.74 MPa, respectively, whereas the maximum von Mises stress of the cortical bone at the distal end of the overdenture of the ball attachments and the resilient telescopic crowns models was 7.82 and 7.06 MPa, respectively.

**Fig. 9:**
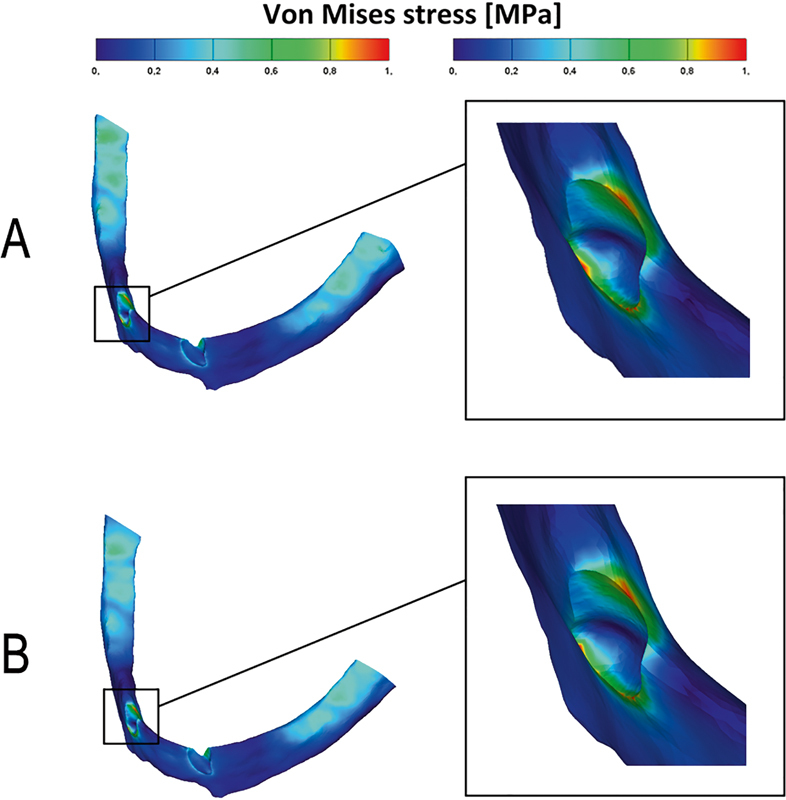
The von Mises stress distribution in the cancellous bone: ball attachments model ( **A** ); resilient telescopic crowns model ( **B** ).

Fig. 9 shows that von Mises stresses of the cancellous bone of the two models were similarly distributed. The von Mises stress concentration areas were around the canines. As illustrated in Fig. 7 , the maximum von Mises stress of the cancellous alveolar bone of the canines of the ball attachments and the resilient telescopic crowns models was 1.29 and 1.05 MPa, respectively.

## Discussion

The RPD with inappropriate design that is unable to distribute chewing forces evenly on the abutment teeth and alveolar bone causes abutment loosing, injury of mucosa, and more absorbing of alveolar process. 14 Therefore, overdenture attachment systems that allow for a better load distribution between the abutments and the alveolar process must be chosen.

The stresses caused by denture during function in oral tissues, such as the abutment teeth and the alveolar bone, cannot be directly measured *in vivo* . 12 26 Experimental methods for stress analysis include electrical strain gauges and photoelasticity. Each method has its limitations, which make it necessary to use two or more methods to analyze the stress and strain in structure of interest. 15 The main disadvantage of strain gauges is that strain measurement is limited in the gauge area, which may not include the area of interest. 33 The photoelasticity method allows stresses to be quantified throughout a 3D structure and identifies stress gradients. Its disadvantage is the requirement for a model of a birefringent material and it is so difficult with complex geometries. 19 FEA is a useful method for investigation and analysis of complex structures that are difficult to standardize during *in vitro* and *in vivo* studies. 18

In this study an accurate method for constructing mucosa FEA model with accurate thickness and morphology was used.

This method depended on fitting the CBCT data of mandible with the scanning data of patient mandibular master model by aligning the matched points of each images then subtracting the mandible bone model from aligned models, so mucosa model with accurate features was gotten. 14 The canines models were designed with Exocad, not constructed from CBCT data of mandible, because the differences of contrast between the canines roots and cancellous bone in the CBCT data was not so significant. The tooth was assumed to be dentine material, as the mechanical property of the enamel and dentine are proven to be similar. 34 A vertical bilateral load of 120 N was applied to the central fossa of first molars. 14 32 For studying the mechanical efficiency of the denture, masticatory loads range from 20 to 90 N can be applied, whereas the maximum masticatory load values can be up to 122 N. 12 The direct bite force is more important compared with the other occlusal load patterns because of the magnitudes, 35 so the vertical load was applied.

The maximum von Mises stress of the canine was concentrated at the buccal cervical region, which corroborates the result found in a study by Kumar et al, 36 where they analyzed the stress of the primary abutments of RPD for class | partially edentulous mandibular arch. The maximum von Mises stress of the cortical bone around the canine was concentrated at the cervical region. This result is similar to that found by Tanaka et al and Pan et al. 37 38

Bone overload leads to resorption, whereas no load over the bone induces atrophy and loss of bone. 39 The level of stress associated with bone resorption has not been clearly established in the literature. 26 The stress-bearing limit of cortical bone has been reported as 170 to 190 Mpa. 29 Whereas the ultimate strength of the cortical bone is 90 Mpa. 40 The values obtained in this study were well below the bearing limit as well as the ultimate strength of cortical bone. The maximum von Mises stress values of the cortical bone were much higher than that of the cancellous bone. This can be attributed to the higher elastic modulus of the cortical bone compared with the cancellous bone. 41 This agreed with many studies. 1 41 42

Ball attachments transferred less stress to the canines and the cortical alveolar bone of the canines than resilient telescopic crowns, but transferred more stress to the cortical bone of alveolar process at the distal end of the overdenture. Ball attachment allows free movement in several directions while resilient telescopic crowns allow free vertical movement. It also allows rotation of the distal end of the overdenture toward the supporting tissues. Resilient telescopic crowns distributed the stresses on the overdenture supported alveolar edge better than ball attachments. The stress generated by the retention systems for overdentures is distributed between the abutment teeth and the alveolar ridge, according to the rigidity of these system. 33 43 Retention systems that allow rotational movements relieved most of the stress of abutment teeth, which is transmitted to the alveolar ridges. 26 43 The more rigid retention systems cause more stress transmitted to the abutting tooth and lower stress concentrated in the edentulous ridge. 44

Ball attachment transferred more stress to the cancellous alveolar bone of the canines. Li et al analyzed the stress of restored root with titanium post, and found that stress passed to root dentin directly from tooth crown to root dentin, and from post to root dentin. 30 The post of the cap of the ball attachment transmitted the stresses in axial direction of the canine root and transferred more stresses apically to the cancellous bone.

## Limitations of the Study

This study has several limitations. The first limitation is that the masticatory force was simulated by applying a vertical bilateral load, while it is important to study other forms of masticatory forces such as oblique loads and unilateral loads. Another limitation is that the living tissues were assumed as isotropic and homogeneous, though the tissues behavior is not. The FEA cannot realistically simulate the tissues behavior, but can reproduce it approximately and give predictive results.

In addition, the FEA is a numerical mathematical solution method, with possible numerical errors. Therefore, randomized clinical studies on this topic must be performed to get the accurate and final results.

## Conclusion

- Overdenture with resilient telescopic crowns distributes the stresses between the canines, alveolar bone of canines, and the overdenture supporting alveolar edge, so it is preferred and indicated when abutment teeth have good periodontal support.

- Overdenture with ball attachments transfers less stress to the canines and cortical alveolar bone of the canines, but more stress to the cancellous alveolar bone of canines and cortical alveolar bone at distal end of the overdenture, so it is indicated with median periodontal-supported abutments.
